# Structure of a Pheromone Receptor-Associated MHC Molecule with an Open and Empty Groove

**DOI:** 10.1371/journal.pbio.0030257

**Published:** 2005-07-12

**Authors:** Rich Olson, Kathryn E Huey-Tubman, Catherine Dulac, Pamela J Bjorkman

**Affiliations:** **1** Division of Biology, California Institute of Technology, Pasadena, California, United States of America,; **2** Howard Hughes Medical Institute, California Institute of Technology, Pasadena, California, United States of America,; **3** Department of Molecular and Cellular Biology, Howard Hughes Medical Institute, Harvard University, Cambridge, Massachusetts, United States of America; University of California, San FranciscoUnited States of America

## Abstract

Neurons in the murine vomeronasal organ (VNO) express a family of class Ib major histocompatibility complex (MHC) proteins (M10s) that interact with the V2R class of VNO receptors. This interaction may play a direct role in the detection of pheromonal cues that initiate reproductive and territorial behaviors. The crystal structure of M10.5, an M10 family member, is similar to that of classical MHC molecules. However, the M10.5 counterpart of the MHC peptide-binding groove is open and unoccupied, revealing the first structure of an empty class I MHC molecule. Similar to empty MHC molecules, but unlike peptide-filled MHC proteins and non-peptide–binding MHC homologs, M10.5 is thermally unstable, suggesting that its groove is normally occupied. However, M10.5 does not bind endogenous peptides when expressed in mammalian cells or when offered a mixture of class I–binding peptides. The F pocket side of the M10.5 groove is open, suggesting that ligands larger than 8–10-mer class I–binding peptides could fit by extending out of the groove. Moreover, variable residues point up from the groove helices, rather than toward the groove as in classical MHC structures. These data suggest that M10s are unlikely to provide specific recognition of class I MHC–binding peptides, but are consistent with binding to other ligands, including proteins such as the V2Rs.

## Abstract 2

MHC-like protein M10.5 is expressed in the vomeronasal organ. The structure does not bind endogenous class I-binding peptides, but is thought to interact with a larger V2R pheromone receptor.

## Introduction

In most mammals, chemical communication between conspecific animals is involved in initiation of reproductive and territorial behaviors. The detection of these species- and gender-specific chemical cues, also called pheromones, is thought to involve receptors of the vomeronasal organ (VNO), a small neuronal epithelium located between the nasal cavity and the palate [1]. Although neurons of the main olfactory epithelium involved in odorant detection ultimately project to cognitive regions of the brain, vomeronasal neurons send inputs via the accessory olfactory bulb to specialized centers of the hypothalamus and amygdala, where they elicit neuroendocrine responses and behaviors such as oestrous synchronization, aggression, and sex discrimination [1–3].

Pheromone receptors belong to the ubiquitous family of G protein–coupled receptors (GPCRs), but are unrelated in sequence to main olfactory epithelium receptors that detect volatile odorants [4,5]. Mouse pheromone receptors can be divided into two subtypes, V1R and V2R, each of which is expressed in the dendritic tips of bipolar neurons in spatially distinct regions of the VNO. The human orthologs of most of these genes appear to be pseudogenes [1]. Mouse V1R receptors are found in the apical VNO domain, are thought to signal through the G-protein α-subunit Gα_i2_, and exhibit sequence similarity to the T2R family of bitter taste receptors [6]. V2R receptors, in contrast, are found in the basal VNO domain, are likely to signal via the G_αo_ molecule, and are related in sequence to metabotropic glutamate (mGluRs), GABA_B_ (γ-aminobutyric acid-B), and calcium sensing receptors. V1R and V2R receptor family members, like all G protein–coupled receptors, contain seven putative transmembrane helices, but, in addition, V2R members include a large N-terminal extracellular domain.

Recently, it was shown that the V2R class of pheromone receptors specifically interacts with members of the mouse M1 and M10 families [7] of major histocompatibility complex (MHC) class Ib proteins [8], which do not appear to have human orthologs [9]. Classical class I MHC molecules, which exhibit high polymorphism in mice, humans, and other mammals, present peptides derived from cytoplasmic proteins to T cells during immune surveillance, and are expressed on most or all nucleated cells [10]. The less polymorphic non-classical class Ib molecules are expressed on a more limited subset of cells and are involved in a variety of functions, including presentation of hydrophobic peptides (e.g., by Qa-2), presentation of formylated peptides by H2-M3, and lipid presentation by CD1 proteins [11]. The non-classical M1 and M10 proteins are expressed exclusively in the VNO and appear to facilitate cell surface expression of V2Rs. Male mice that are genetically deficient in the class I MHC–associated β2-microglobulin (β2m) light chain show no surface expression of V2R pheromone receptors in the dendritic terminals of VNO sensory neurons and lack aggressive behavior toward intruder males [8]. Facilitation of V2R surface expression appears to involve M10 binding to a V2R because immunopurification of VNO receptors identified a multimolecular complex formed between M10, β2m, and a V2R protein. A given neuron generally expresses only one of the roughly 150 different subtypes of V2R receptor and one of the nine known VNO–specific MHC class Ib proteins (six M10 and three M1 family genes) [8,12]. Genetic analysis of individual neurons suggests specificity in binding between different V2R and M10 proteins [8].

Homologous receptors to the V2R proteins, such as the GABA_B_ receptors and the umami and sweet taste receptors, also require accessory proteins for effective surface expression [13–15]. However, the nature of M10s as MHC molecules together with the association between the MHC and mating behavior in rodents [16] offers the intriguing possibility that M10 proteins play a direct role in mate or species recognition. Mice tend to choose mates with disparate MHC haplotypes [17], and pregnant mice have a higher incidence of spontaneous abortion if exposed to the scent of a male with a different MHC haplotype (the Bruce effect) [18]. These functions, as well as other species-specific indicators, may be mediated via M10-V2R complexes, perhaps by binding peptides or other accessory molecules in the canonical peptide-binding groove that exists in classical class I MHC structures [10]. Peptide binding during heavy and light chain assembly in the endoplasmic reticulum (ER), as occurs for classical class I MHC molecules, seems unlikely for M10 proteins because in situ hybridization assays demonstrate that TAP1 and TAP2, which are required for transport of cytoplasmic-derived peptides into the ER, are not expressed in the VNO [8]. However, a recent study shows that V2R–containing neurons are activated by nonameric class I MHC–binding peptides [19], suggesting involvement of exogenously acquired MHC–binding peptides, and perhaps M10 proteins, in individual mate or species recognition.

To determine the peptide-binding capability and structural characteristics of V2R receptor-associated MHC molecules, we expressed, characterized, and solved the crystal structure of the ectodomain of M10.5, an M10 family member. The M10.5 structure reveals an open conformation of the α1–α2 domain helices that contains no ordered peptidic or non-peptidic occupant, which represents the first example, to our knowledge, of the structure of an empty class I MHC molecule. Thermal stability studies suggest that the M10.5 groove is normally occupied; however M10.5 does not associate with the sorts of peptides that normally bind to class I MHC molecules. These results suggest a new and divergent function for the M10 family of proteins.

## Results

### The M10.5 Structure

A soluble form of M10.5 was expressed together with human β2m in baculovirus-infected insect cells, and soluble M10.5–β2m complexes were purified from cell supernatants. Similar efforts to express soluble M10.5 together with mouse β2m in insect cells or Chinese hamster ovary (CHO) cells were unsuccessful (data not shown), perhaps related to the observation that mouse class I MHC molecules form stronger heterodimeric complexes with human, as compared with mouse, β2m [20,21]. M10.5 was crystallized in space group P2_1_2_1_2_1_ with five molecules in the asymmetric unit. Most crystals diffracted weakly to 3.5–4.0 Å resolution, but an incomplete dataset from a rare crystal that diffracted beyond 3.5 Å was combined with a more complete dataset to 4.0 Å (Table 1). The structure was solved by molecular replacement using the mouse class Ib MHC molecule Qa-2 [22] as a search model. Refinement using non-crystallographic symmetry (NCS) restraints yielded a final model (*R*_cryst_ = 26.6%; *R*_free_ = 30.3%) (Table 1). Although data to 3.0 Å were included in the refinement, the high-resolution data are incomplete, thus the effective resolution of the structure is 3.2 Å. Two loops comprising residues 145–150 (α2 domain) and 194–197 (α3 domain) are missing in electron-density maps and are not included in our model.

**Table 1:**
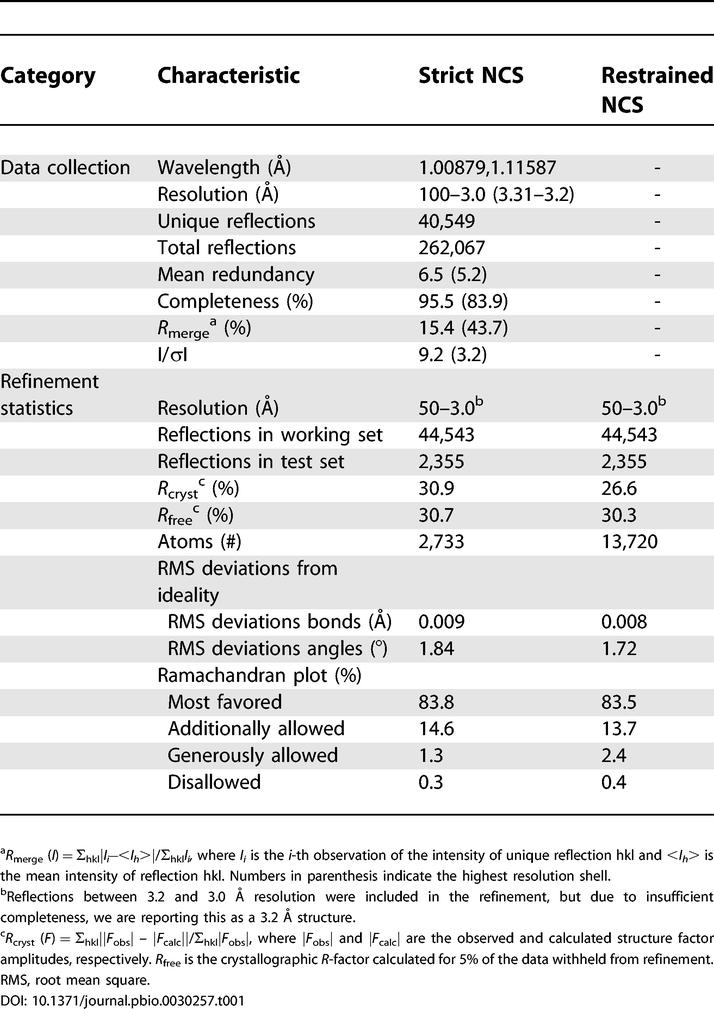
Crystallographic Data and Refinement Statistics

**Figure 1:**
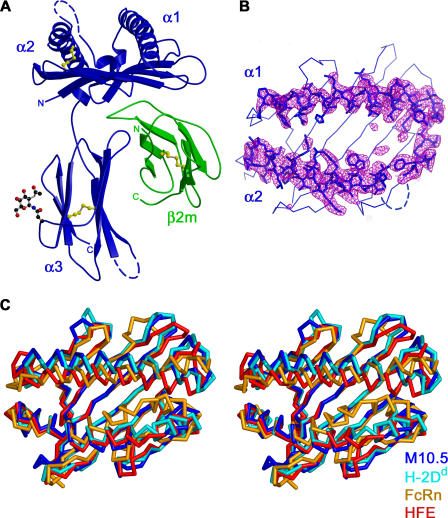
The Structure of M10.5 (A) Ribbon diagram of M10.5 (side view). The heavy chain is blue, the β2m light chain is green, disulfide bonds are yellow, and ordered carbohydrate attached to Asn223 is shown in ball-and-stick representation. Two disordered loops in the heavy chain are shown as dashed blue lines. (B) Top view of the α1–α2 platform overlaid with an F_o_-F_c_ annealed omit electron-density map contoured at 3.5 σ. The map was calculated for one of five molecules in the asymmetric unit using NCS restraints in the annealing process. Residues 55–84 and 137–174, shown in stick representation, were omitted from the structure factor calculation. Electron density is absent for residues 145–150 (dashed line), indicating that they are disordered. No significant electron density is observed in the groove between the α1 and α2 helices. (C) Stereo view of the superposition of the α1–α2 platforms from M10.5, H-2D^d^ [24], FcRn [32], and HFE [33]. Structures were aligned using residues classified as platform β-sheet residues (see Materials and Methods). The cleft between α1 and α2 helices is significantly narrower in FcRn and HFE than in M10.5 and H-2D^d^.

The overall structure of M10.5 resembles the structures of classical class I MHC molecules. A BLAST search [23] identifies mouse H-2D^d^ as the most closely related classical class I MHC molecule (approximately 50% amino acid identity) for which a structure is available [24], thus we have used H-2D^d^ for comparisons. As in other class I structures, the first 180 residues of the M10.5 heavy chain form the α1–α2 domain superdomain, which is composed of an eight-stranded anti-parallel β-sheet platform topped by two anti-parallel α-helices. The following approximately 90 residues form the α3 domain, which resembles an immunoglobulin constant region domain (Figure 1A and 1B). The non-covalently attached β2m light chain, also resembling an immunoglobulin constant region, contacts both the underside of the α1–α2 platform and one β-sheet of the α3 domain in an orientation consistent with previously solved mouse and human class I MHC structures, indicating that pairing human β2m with the mouse M10.5 heavy chain does not disrupt the overall M10.5 structure.

Ordered N-linked carbohydrate is observed attached to Asn223 within the loop joining the third and fourth β-strand in the α3 domain. The carbohydrate occludes the counterpart of the region in class I MHC molecules that is the major determinant for binding the T cell co-receptor CD8 [25], likely preventing M10.5 from participating in CD8^+^ T cell–mediated immunological responses. Ordered carbohydrate is not observed at either of the two other predicted N-linked glycosylation sites (Asn62 and Asn198), but the quality of the electron-density map is poor in these regions.

### The α1–α2 Platform of M10.5 Contains an Open, but Apparently Empty, Groove

A large groove between the α1 and α2 domain helices forms the peptide-binding site in classical class I MHC molecules [10]. Structural studies of class I MHC molecules and homologs show a correlation between the degree of separation of the α1–α2 domain helices and the ability to bind peptides or other small molecules [26]. The helices are separated by approximately 18 Å in the center of the grooves of classical peptide-binding class I MHC molecules such as H-2D^d^ [24], and class I MHC-related proteins that bind other small molecule ligands also contain open grooves with separated α1 and α2 domain helices [27–31]. In contrast, the neonatal Fc receptor (FcRn) [32], the hemochromatosis protein HFE [33], and MIC-A [34], MHC homologs that do not bind small molecule ligands, have collapsed grooves with a smaller separation between the α1–α2 domain helices [32–34].

A superposition of the α1–α2 platforms of M10.5, H-2D^d^, FcRn, and HFE illustrates the variation in groove size (Figure 1C) and demonstrates that M10.5 has an open groove more similar to the peptide-binding classical class I MHC molecules than the non–peptide-binding homologs. The M10.5 α1–α2 domain helices superimpose well with the analogous H-2D^d^ helices (RMS [root mean square] deviation of 1.22 Å for 164 of 181 α-carbon atoms), with the largest differences located in the region of the α2 domain helix immediately preceding six residues that are disordered in M10.5 (residues 145 to 150). The overall similarity of the M10.5 and H-2D^d^ α1–α2 platforms, which are contained within class I heavy chains that are paired with human (M10.5) or mouse (H-2D^d^) β2m light chains, demonstrates that the observed structure of the M10.5 α1–α2 platform does not result from an artifactual change induced by pairing with human, rather than mouse, β2m. The more open character of the M10.5 groove as compared with the grooves of non–peptide-binding MHC homologs is seen when the calculated accessible surface areas are compared: approximately 730 Å^2^ for M10.5 compared with approximately 760 Å^2^ for typical class I MHC molecules [30,33], approximately 690 Å^2^ for H-2D^d^, and approximately 415 Å^2^ and approximately 235 Å^2^ for HFE and FcRn, respectively [33] (see Materials and Methods). Thus the M10.5 groove can be classified as “open” and capable of binding to a ligand.

The α1–α2 domain groove is occupied by a peptide or mixture of peptides in all class I MHC structures solved to date, and continuous electron-density representing peptide(s) is always seen in the α1–α2 groove [35]. Indeed, attempts to crystallize an empty version of H-2K^b^, which was expressed in cells lacking peptide-loading machinery and purified in the absence of a binding peptide, resulted in a crystal structure that revealed a peptide derived from the cell growth media [36]. M10.5 omit electron-density maps in which the α1 and α2 helices were removed from structure factor calculations return clear density for the omitted region (Figure 1B), indicating that the maps are of sufficient quality to detect bound molecules of the size of an 8–10 residue peptide. However, M10.5 electron-density maps show no ordered density corresponding to a peptide: Annealed F_o_-F_c_ maps calculated using NCS constraints or tight restraints do not show continuous electron density within the groove (Figure S1), and maps calculated before or after 5-fold real space averaging also failed to reveal unbroken density in the cleft (data not shown). The possibility that a minor peak near the center of the groove in averaged maps represents a portion of a bound polyethylene glycol molecule is discussed in the caption of Figure 1. We conclude that the M10.5 groove does not contain a single defined peptidic or non-peptidic occupant or a mixture of compounds with a similar conformation.

### Comparison of M10.5 and Classical Class I MHC Grooves

**Figure 2:**
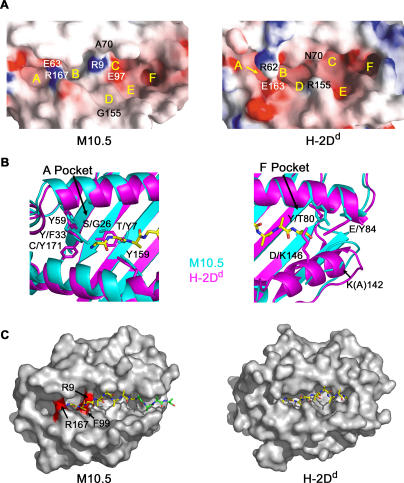
Groove Surface Characteristics (A) Electrostatic surface representations calculated using GRASP [42] of the M10.5 (left) and H-2D^d^ (right) grooves. Positions of the six pockets are labeled A–F in yellow. In H-2D^d^, Arg62 and Glu163 create a bridge over the peptide-binding groove. In M10.5, Glu63 and Arg167 define a similar feature. The H-2D^d^ groove is constricted between Asn70 and Arg155. In M10.5, Ala70 and Gly155 lead to a wider groove and continuous D and E pockets. (B) Comparison of residues in the A and F pockets of classical class I MHC molecules and M10.5. All alanine-peptide (yellow, atoms color-coded according to atom type) is derived from the H-2D^d^ structure [24]. Left: In class I MHC proteins, a cluster of four tyrosine residues in the A pocket (Tyr7, Tyr59, Tyr159, and Tyr171) form hydrogen bonds with backbone atoms in the peptide N-terminus (residues are listed in single letter code for M10.5 and H-2D^d^, respectively). In M10.5, Tyr7 is replaced by a threonine residue and Tyr171 is replaced with a cysteine. Five of the six M10 proteins have an additional tyrosine at position 33 that could potentially replace one of the two missing tyrosine residues. M10.5 has a serine instead of a glycine at position 26, which could participate in additional hydrogen-binding interactions. Right: The F pocket of class I MHC molecules is blocked on one end by Thr80, Tyr84, and Lys146. The M10.5 pocket is open on this end (see panels A and C) due to the substitution of a glutamate residue for Tyr84 and the disorder of the loop containing residue 146 (Asp in M10.5). Lys142 in M10.5 is missing sidechain density and has been modeled as an alanine, but most likely would not further occlude the M10.5 F pocket. (C) Molecular surface representations of M10.5 and H-2D^d^ with an all-alanine peptide (yellow) derived from the H-2D^d^ structure [24] superimposed in the M10.5 groove. M10.5 atoms that came within 2.5 Å of the peptide trace were considered clashes and are colored red. Four additional alanine residues (green) were added to the C-terminus of the H-2D^d^–binding peptide to illustrate that peptides binding in the M10.5 groove could extend out of the F pocket side.

Crystallographic studies of classical class I MHC–peptide complexes have defined six pockets (A–F) within the peptide-binding groove that interact with various portions of bound peptides (Figure 2A) [37–39]. The A and F pockets at either end of the peptide-binding groove are largely conserved and interact with the N- and C-terminus, respectively, of the bound peptide [37,39]. The B, C, D, and E pockets contain residues that vary between alleles, resulting in different allele-specific peptide-binding preferences.

The A and F pocket regions of the M10.5 groove contain substitutions that prevent the interactions that anchor peptide termini into the groove of a classical class I MHC molecule. Two of the four conserved tyrosines in the MHC class I A pocket (tyrosines 7, 59, 159, and 171), which hydrogen bond directly or through a water molecule with main chain atoms of the peptide N-terminal residue [37,39], are substituted in M10.5 as Thr7 and Cys171 (Figure 2B). Substitutions in other M10s also eliminate one or two of the four A pocket tyrosines [8,12]. It remains possible, however, that M10.5 and other M10s could bind a peptide N-terminus in a non-classical manner, utilizing residues including Thr7, Ser26, and Tyr33 as hydrogen-bond donors. Residues that typically anchor the C-terminus of a peptide in the class I MHC F pocket are also different in M10.5. In H-2D^d^, the ninth and tenth residue of the bound peptide (P9 and P10) form hydrogen-bonding and van der Waals contacts with Val76, Asp77, Tyr84, Thr143, Lys146, and Trp147 (Figure 2B). In M10.5 and most other M10s, these residues are replaced by non-conservative substitutions, and the last two are missing entirely from the M10.5 electron-density map. Thus potential interactions between M10 F pocket groove residues and peptides would have to occur in a non-classical manner.

An interesting feature of possible functional relevance is the relatively open nature of the F pocket side of the M10.5 groove (Figure 2C), suggesting that potential small molecule or peptide occupants could extend out of this side of the groove. Homology models of other M10 proteins constructed using the M10.5 structure and the Swiss-model Protein modeling Server [40] suggest that this open character is a general feature of M10 proteins (data not shown). In class I MHC molecules, Tyr84 and Lys146 occlude the F pocket side of the groove, but the tyrosine to glutamate substitution at position 84 in M10.5 (Figure 2B) contributes to the more open M10.5 groove. In addition, M10.5 Asp146 is part of the disordered 145–150 region of the α2 domain helix, thus it does not contribute to closing the F pocket side of the groove. The disorder of these residues may be related to a need to maintain flexibility in the F pocket end of the M10.5 groove to allow binding of ligands that extend out of the M10.5 groove.

**Figure 3:**
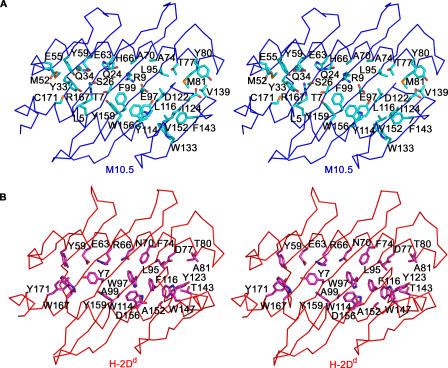
Groove Residues in M10.5 and H-2D^d^ Stereo view of residues lining the α1–α2 groove in M10.5 (A) and H-2D^d^ (B). Groove residues are defined as in Table 2.

In order to make additional comparisons between the grooves of M10.5 and peptide-binding class I MHC molecules, we used a previously described algorithm [30,33] to identify residues that comprise the grooves of M10.5 and H-2D^d^. Residues that line the M10.5 and H-2D^d^ grooves were defined as having more than 5.0 Å^2^ accessible surface area to a 1.4 Å probe and less than 5.0 Å^2^ to a 5.0 Å probe (Table 2, Figure 3A and 3B), resulting in 33 groove residues in M10.5 and 22 groove residues in H-2D^d^. The additional groove residues in M10.5 that are buried in H-2D^d^ are divided into two clusters, one in the area surrounding the A and B pockets, and the other around the F pocket (see Figure 2A). The M10.5 A pocket is larger than its H-2D^d^ counterpart due to the substitution of class I MHC residues Tyr7 and Tyr171 with less-bulky threonine and cysteine residues, respectively, and a different location of the sidechain of M10.5 Arg167 as compared to H-2D^d^ Trp167. Arg167 was postulated to likely block peptide binding in M10.5 [8] by analogy to Arg167 in FcRn, which partially occludes the A-B pocket [32]. However, the sidechain of Arg167 in M10.5 is partially disordered, suggesting mobility that could result in a rearrangement to allow a groove occupant to access the open pocket area below the arginine sidechain. Other differences that create a larger M10.5 A pocket compared to H-2D^d^ involve several large aromatic residues lining the H-2D^d^ groove that are replaced by smaller non-aromatic residues in M10.5. These include H-2D^d^ Tyr7, Phe74, Trp97, Phe116, Trp167, and Tyr171, which are substituted as Thr7, Ala74, Glu97, Leu116, Arg167, and Cys171 in M10.5. Another difference between the M10.5 and H-2D^d^ grooves is a considerable enlargement of the M10.5 D pocket, which is continuous with the E pocket. The enlargement is caused by the replacement of H-2D^d^ residues Asn70 and Arg155 with alanine and glycine residues, respectively, removing the constriction that sandwiches the peptide residue at position 4 (glycine) in the H-2D^d^ structure [24].

**Table 2:**
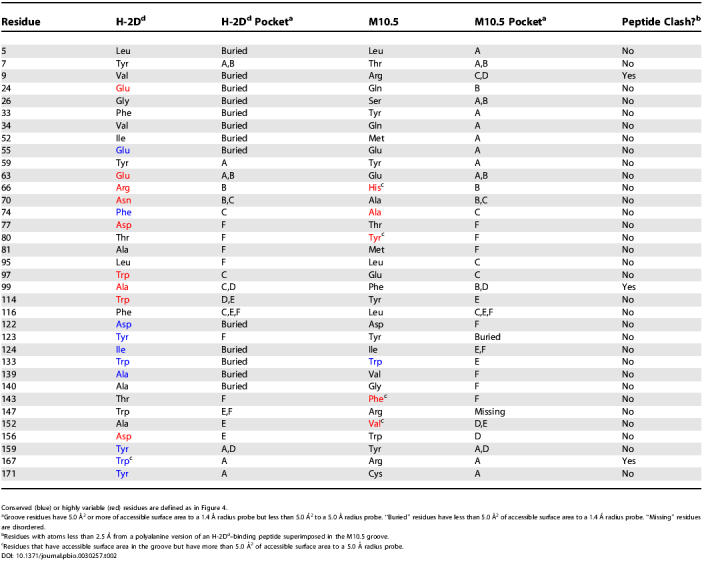
Groove Residues

The chemical character of the groove of MHC–related protein can sometimes reveal the nature of its ligand. For example, the largely hydrophobic grooves of CD1 [30] and the class Ib MHC molecules Qa-2 [22] and HLA-E [41] allow binding of lipids (CD1) and hydrophobic peptides (the class Ib proteins). By contrast, the M10.5 groove, like the grooves of H-2D^d^ and other classical class I MHC molecules, contains a mixture of polar and non-polar residues (Table 2). A surface potential map generated by GRASP [42] shows that the A, C, E, and F pockets of M10.5 are slightly acidic whereas the B and D pockets are uncharged (see Figure 2A). The sidechain of Arg9 (a buried valine in H-2D^d^) points into the center of the M10.5 groove and forms a salt-bridge with the sidechain of Glu97. A second salt-bridge between Glu63 and Arg167 bridges the A and B pockets. The groove in H-2D^d^ is primarily acidic, with a similar salt bridge formed between Arg62 and Glu163 over the A–B pocket boundary (Figure 2A). Peptides bound to H-2D^d^ are accommodated under the salt bridge [24], thus the salt bridge in the M10.5 groove would not necessarily prevent binding of a peptide or other small molecule.

### Sequence Conservation and Receptor Binding

**Figure 4:**
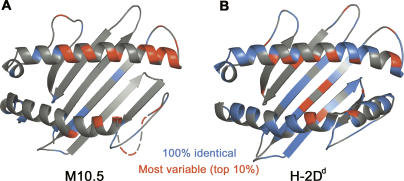
Sequence Conservation in M10 and H-2 Loci (A) Ribbon diagram of the M10.5 α1–α2 platform with positions of residues that are 100% identical in the nine M10 and M1 families colored blue; residues that are among the top 10% most variable are colored red. Variability is determined by the number of amino acids at a given position divided by the frequency of the most common allele at that position. Residues 145–150, which are disordered, are designated by a dashed line. (B) Ribbon diagram of the H-2D^d^ α1–α2 domain. Conserved (blue) and highly variable (red) residues are derived from an alignment of 19 H-2D alleles [80].

Like classical class I MHC molecules, variability within M10 proteins is mainly localized to the α1–α2 platform, with the α3 domain being more constant [8,12]. As previously predicted [8] and now confirmed by the M10.5 structure, amino acids with the highest degree of sequence variability within the M10 and M1 families cluster on the top face of the α1 and α2 domain helices. Indeed, three of the six disordered residues (145–150) in the M10.5 α2 domain helix belong to the top 10% of variable residues within the M10 α1 and α2 domains. By contrast, M10 residues that point into the groove show less variability (Figure 4A). This pattern of variability is opposite to the pattern in classical MHC class I molecules, in which residues that display the greatest sequence variability point towards the peptide-binding groove, resulting in allelic specificity for binding peptides, whereas residues on the tops of the α1 and α2 domain helices are more conserved [43,44] (Figure 4B).

### M10.5 Is Thermally Unstable

Classical class I MHC molecules and UL18 are thermally unstable in the absence of bound peptide [21,45,46]. In contrast, non-peptide binding class I MHC homologs, such as FcRn and the hemochromatosis protein HFE, do not show reduced thermal stability, presumably due to structural rearrangements that close the counterparts of their peptide-binding grooves [32,33,47]. The M10.5 groove is open, but apparently unoccupied, in the crystal structure, raising the question of whether M10.5 is stable in the absence of a groove occupant.

**Figure 5:**
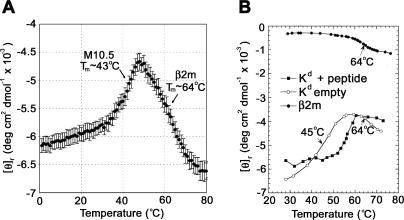
Thermal Denaturation Profiles Thermal denaturation of M10.5 (A) compared with empty and peptide-filled H-2K^d^ (B) (modified from Figure 3A in Fahnestock et al. [21]). The CD signal at 223 nm was monitored as a function of temperature for insect cell-derived M10.5 and empty and peptide-filled versions of soluble H-2K^d^ produced in CHO cells. *T*_m_s for the melting of the M10.5 heavy chain and β2m light chain (marked with arrows) were derived by estimating the half-point of the ellipticity change between the beginning and end of each transition. The M10.5 denaturation profile is similar to the profile obtained from empty H-2K^d^, suggesting that the M10.5 groove is not occupied. The possibility that polyethylene glycol, a component in the crystallization solutions, might bind to and stabilize M10.5 was investigated by repeating the unfolding experiment in the presence of 20% polyethylene glycol 1000, with no significant changes to the thermal stability profile (data not shown).

To determine the thermal stability of M10.5, we monitored heat-induced unfolding by recording the circular dichroism (CD) signal at 223 nm as a function of increasing temperature. Two unfolding transitions, an upward-sloping transition with a T_m_ of 43 °C and a downward-sloping transition with a T_m_ of 64 °C, are evident in the melting curve of insect cell-derived M10.5 (Figure 5A). The M10.5 melting curve and derived T_m_ values are similar to previously reported results derived from an empty form of the mouse class I MHC molecule H-2K^d^ complexed with human β2m [21,46]. In these studies, the first transition (T_m_ = 43–45 °C) represented the unfolding of the empty H-2K^d^ heavy chain, and the second transition (T_m_ = 64 °C) represented the independent unfolding of β2m subsequent to heavy chain denaturation [21,46] (Figure 5B). By analogy, we interpret the first and second transitions in the M10.5 melting curve as resulting from M10.5 and β2m denaturations, respectively. In contrast to the low thermal stability of M10.5, the peptide-filled form of the H-2K^d^ heavy chain melts with a T_m_ of approximately 57 °C [21] (Figure 5B) and FcRn melts with a T_m_ of approximately 62 °C at pH 6 [47]. The low thermal stability of purified M10.5 suggests that a ligand or binding partner not present in the purified preparation is necessary to stabilize the native conformation at 37 °C in vivo.

### M10.5 Does Not Associate with Endogenous Peptides

The empty groove in the M10.5 structure could result from expression in invertebrate cells, which do not possess the machinery to load peptides into class I MHC molecules [48,49]. When classical class I MHC molecules are expressed in vertebrate cells or purified from native sources, mixtures of short peptides derived from cytoplasmic proteins can be eluted [50]. Sequence analyses of eluted peptides revealed that any given class I allele can bind a wide variety of short (8- to 10-mer) peptides that conform to a particular allele-specific peptide-binding motif involving preferred residues at the peptide C-terminus and an internal position (usually position 2 or 5/6) [50]. To determine whether M10.5 binds endogenous or exogenous peptides with these or other characteristics, we expressed the M10.5 ectodomain in CHO cells, mammalian cells that support peptide loading, and examined acid eluates from purified M10.5 using methods previously used to identify peptides eluted from class I MHC molecules [51].

Purified M10.5-β2m heterodimers expressed in CHO and in insect cells were treated with acetic acid to dissociate potential peptide material. Insect cell-derived M10.5 was analyzed before and after incubation with a mixture of short (8- to 9-mer) peptides. As it would be impossible to test all possible 8- to 9-mer peptides, we prepared a mixture of peptides including those reported to activate V2R-expressing neurons [19] in order to see if any of these class I MHC-binding peptides bind to M10.5. We used soluble versions of two other β2m-binding class I MHC–like proteins expressed in CHO cells as controls: UL18, a viral class I MHC homolog that binds endogenous peptides resembling class I MHC–binding peptides [52], and FcRn, a class I MHC homolog that does not bind peptides or contain a non-peptidic groove occupant [32,47].

Low molecular weight acid eluates derived from M10.5 and the control proteins were sequenced by Edman degradation and analyzed by MALDI-TOF (matrix-assisted laser desorption ionization time-of-flight) mass spectrometry. Acid eluates from CHO–derived M10.5 and both peptide-treated and untreated insect cell-derived M10.5 resemble eluates from FcRn, the non–peptide-binding class I homolog, rather than eluates from UL18, a peptide-binding protein (Table 3). With the exception of cycle 1, which typically shows a high background for FcRn and other proteins that do not bind peptides [52], the total yield of the amino acids from each cycle of pool sequencing of the M10.5 acid eluates remains nearly constant and not significantly above background. By contrast, the UL18 acid eluate shows the presence of a mixture of peptides with characteristics similar to those previously reported [52]. MALDI-TOF analysis of the acid eluates indicated molecules with masses consistent with peptides in the UL18 sample, but not in the negative control or M10.5 samples (data not shown). Thus M10.5 does not appear to bind any form of peptide, including N-terminally blocked peptides, or other small molecule ligand when expressed in CHO or insect cells. In addition, purified M10.5 does not bind any of a collection of class I MHC–binding peptides, as would have been expected if M10 proteins were the molecules responsible for binding the class I MHC–binding peptides reported to activate VNO neurons [19].

**Table 3:**
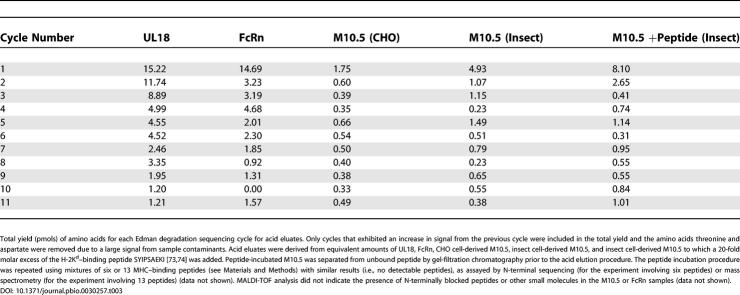
Amino Acid Yields from Acid Eluates

### Computer Modeling of Potential M10.5 Groove Occupants

Given the open character of the M10.5 groove and the thermal instability of M10.5-β2m heterodimers, we believe that the groove is likely to be a binding site for a peptide, protein, or a non-peptidic small molecule ligand. Here we use computer modeling to evaluate which potential ligands can fit into the M10.5 groove.

We first consider whether the M10.5 groove can accommodate a class I MHC–binding peptide by superimposing a polyalanine version of an H-2D^d^–binding peptide [24] or a Qa-2–binding peptide [22] on the M10.5 structure (see Figure 2C). The H-2D^d^–binding peptide fits into most regions of the M10.5 groove, but clashes with three residues in the A and B pockets (see Table 2). The Qa-2 peptide adopts a sharper upward bend near residue P2 (data not shown), and consequently, two of these clashes (Arg9 and Phe99) do not occur. The third clash involves Arg167 with the N-terminus of the peptide, but arginine sidechains are often flexible and might be able to move out of the way of the N-terminus of a bound peptide. Several residues in the vicinity of Arg167 have smaller sidechains in M10.5 than in H-2D^d^, which could open up additional space for alternative conformations of a peptide terminus. These include Thr7 (Tyr7 in H-2D^d^) and Cys171 (Tyr171 in H-2D^d^), as well as Arg167 (Trp167 in H-2D^d^) if its sidechain can adopt an alternate conformation. We conclude that the M10.5 groove can accommodate a peptide that adopts a class I MHC–binding conformation, but that differences between the A and F pocket regions of M10.5 and classical class I MHC molecules (see Figure 2B) would require a peptide bound in the M10.5 groove to be anchored differently than a class I MHC–binding peptide.

We next considered whether longer peptides or an extended region of a protein could fit into the M10.5 groove by extending the polyalanine version of the H-2D^d^–binding peptide by four alanine residues at its C-terminus. The resulting peptide was fit into the M10.5 groove with the extra residues extending out of the F pocket side of the groove (Figure 2C). The relatively open F pocket end of the M10.5 groove is able to accommodate the extra residues, suggesting that M10 proteins could bind extended regions of other proteins. Candidate M10–binding proteins include the V2Rs and/or the major urinary proteins (MUPs), which are thought to deliver small-molecule compounds to chemosensory receptor neurons [1,12]. Although the nature of the interaction is unknown, biochemical data demonstrate an association between M10 and V2R proteins [8], thus the open M10.5 groove may form a binding site for a piece of a V2R. MUPs, however, are not known to associate with M10 proteins, and a surface plasmon resonance-based binding experiment did not reveal an interaction between immobilized M10.5 and MUPs in fractionated mouse urine (data not shown; see Materials and Methods).

It is also possible that pheromones bind within the M10 groove and either are presented directly to V2R receptors or initiate a conformational change within the M10 molecule that results in the activation of the receptor. Because there are no known V2R–activating ligands, we examined pheromones known to activate V1R receptors including 2-heptanone, α- or β-farnesene, and 2,5-dimethylpyrazine [53]. Although these molecules fit easily within the M10.5 groove (data not shown), the hydrophobic nature of most pheromones is not complementary to the charged character of the groove (see Figure 2A), which is much larger than a single pheromone molecule. Smaller pheromones might associate with residues within the relatively hydrophobic M10.5 D pocket (Figure 2A), however, the rest of the groove would remain unoccupied and presumably still unstable, and it is not obvious how such an interaction would lead to activation of a V2R if it occurs through a conformational change in an M10 groove.

## Discussion

The M10 family of murine class Ib MHC molecules are expressed exclusively in the VNO and appear to act as chaperones to facilitate cell surface expression of the V2R class of pheromone receptors [1,8]. Although the role of M10 molecules in V2R signaling is unclear, direct homologs of proteins typically associated with immunogenic identity such as MHC proteins provide attractive candidates to mediate MHC–disassortative mating preferences [17] and pregnancy-block phenomena [18]. Here we present a biochemical and structural analysis of M10.5, a representative M10 molecule, aimed at providing new insights into the function of M10 proteins and their association with V2Rs. As M10 proteins are related by greater than 60% sequence identity [8,12], above the approximately 30% sequence identity threshold suggesting similar three-dimensional structures [54], the results obtained for M10.5 are relevant to other M10 proteins.

Proteins with an MHC fold generally have open, occupied grooves, as in classical MHC proteins, other class Ib proteins, CD1 [30], Zn-α2-glycoprotein [28], and the protein C receptor [55], or closed, unoccupied grooves, as in FcRn [32], HFE [33], MIC-A [34], Rae-1β [56], and T22 [57]. Thus it is surprising that the M10.5 groove is open, but apparently unoccupied, in the crystal structure. Consistent with the lack of defined extra electron density for a groove occupant, analyses of acid eluates derived from M10.5 expressed in insect cells and in CHO cells failed to reveal peptidic or non-peptidic material (Table 3). In addition, M10.5 did not bind MHC–binding peptides, including peptides reported to activate VNO neurons expressing M10 family members [19]. These results do not rule out a peptide-binding role for M10 proteins because the peptides used for these experiments may not have been optimal for binding to M10.5. However, the fact that M10.5 does not associate with any endogenous peptides when expressed in CHO cells suggests that it either has more stringent criteria for peptide binding than conventional class I proteins, which bind endogenous peptides when expressed in CHO cells [21], or that it does not bind peptides at all. If M10.5 does bind peptides, changes in the M10 A and F pockets from their counterparts in class I MHC grooves (see Figure 2B) suggest that M10.5 and other M10 molecules cannot bind the same sort of peptides that are bound by class I MHC molecules. Thus it is unlikely that the class I MHC–binding peptides reported to activate VNO neurons [19] exert their effects by binding to M10 family members, consistent with the observation that purified M10.5 showed no detectable binding to peptides used in that study.

Although the ectodomains of classical class I MHC molecules can fold in the absence of peptide [21,58], and full-length empty class I proteins can reach the cell surface, they are thermally unstable and are rapidly degraded unless an appropriate binding peptide is added exogenously or the cells are grown at 26 °C [45]. Heat-induced unfolding of recombinant M10.5 reveals a thermal stability similar to empty, rather than peptide-filled, class I MHC molecules (Figure 5A and 5B). It has been assumed that the low thermal stability of empty class I MHC molecules contributes to the apparent inability of empty class I molecules to crystallize (unpublished data). Indeed, the results of molecular dynamics simulations have been used to predict that empty α1–α2 platforms do not adopt a single defined conformation [59]. However, in the case of the empty M10.5 α1–α2 platform, we are able to generate moderately well-ordered crystals, and we see a single conformation for the five copies of M10.5 in the crystallographic asymmetric unit (Figure S1). The empty platforms may be stabilized somewhat by crystal contacts, but different crystal contacts for the five M10.5 molecules do not produce different conformations of the α1–α2 platform, as evidenced by relatively low temperature factors for α1–α2 residues (see Materials and Methods).

The low thermal stability of empty recombinant M10.5 suggests that the groove is normally occupied to stabilize the protein at 37 °C. Although our results cannot be used to identify a physiologically relevant groove occupant, the structural and biochemical results can be used to determine which types of ligands are unlikely. Our analysis of the M10.5 structure and the fact that endogenous peptides were not found associated with M10.5 expressed in CHO cells (Table 3) do not support a model in which M10 proteins are stabilized by class I MHC–binding peptides from either endogenous or exogenous sources. In addition, the M10.5 groove does not have the largely hydrophobic character that would be expected if it were a binding site for hydrophobic pheromones serving as chemical cues in urine, and we observed no detectable binding signals when fractionated mouse urine was injected over purified M10.5 in a surface plasmon resonance-based binding assay (data not shown, see Materials and Methods).

A clue as to the possible nature of an M10 groove occupant comes from the different patterns of variability within the grooves of M10 and classical class I MHC proteins (Figure 4), which likely reflect the different functional roles of vomeronasal versus classical MHC molecules. Variability within the grooves of classical class I MHC molecules, which mainly involves inward-pointing groove residues [43], creates different peptide-binding preferences such that different alleles present different types of peptides that conform to allele-specific peptide-binding motifs [35]. If the grooves of M10 proteins are occupied, the relative conservation of residues that point toward the M10 groove suggests that different M10 proteins bind a more limited set of ligands than the ligands of different class I MHC alleles. The greater variability in the upward-pointing residues on the M10 helices (Figure 4A) suggests allele-specific interactions with other proteins, consistent with the suggestion of specificity in binding between different V2R and M10 proteins [8]. The variability analysis combined with the M10.5 structure suggests a potential interaction site: The disordered six-residue loop within the M10.5 α2 domain (residues 145–150) contains three highly variable residues that could be involved in an interaction with a V2R. As a precedent for a disordered region of an MHC-like structure being at a receptor binding site, a portion of the α2 domain helix that is disordered in the structure of MIC-A (residues 152 to 161, corresponding to approximately the same M10.5 residues) [34] becomes ordered in a structure of MIC-A bound to the NKG2D receptor [60].

The disordered region of the M10.5 α2 domain helix may also be related to a need for flexibility within the F pocket region of the M10 groove, which could allow binding of larger ligands than the 8- to 10-mer peptides bound in the grooves of classical class I MHC molecules. Although the M10.5 groove is about the same size as the grooves of classical class I MHC molecules (approximately 730 Å^2^ versus approximately 760 Å^2^), and the A pocket side of both types of grooves is closed, the F pocket side of the M10.5 groove is more open than the counterpart region of a class I molecule (see Figure 2). The closed ends of class I MHC grooves result in the preference for binding short (8- to 10-mer) peptides that do not extend out of either end of the groove [35]. The open F pocket end of the M10.5 groove may allow it to bind larger ligands, perhaps even an extended region of an intact protein, such as a V2R. As a precedent for this type of interaction, the open grooves of class II MHC molecules bind to an extended region with the invariant chain protein during transit through the ER and Golgi (reviewed in [61]). In this case, the invariant chain not only occludes the class II MHC groove to prevent association with ER peptides, it also serves as a chaperone to direct class II proteins to acidic compartments where they acquire peptides derived from exogenous proteins. A related situation could occur for M10 proteins, such that an extended region within the ectodomain of a V2R would bind within the groove of an M10 protein during transit through the ER and Golgi. An interaction in which an extended loop from a V2R protein binds to an M10 groove would explain why peptides or other small molecule ligands were not found in recombinant M10.5 molecules expressed in the absence of V2R proteins and why the empty recombinant molecules are thermally unstable. In this hypothesized scenario, newly synthesized M10 and V2R proteins would be stabilized through mutual interactions with a V2R loop in the M10 groove, enabling the M10 to escort the V2R to the cell surface, rationalizing the observation that M10 proteins are required for cell surface expression of V2Rs [8].

## Materials and Methods

### M10.5 expression and purification

A construct encoding soluble M10.5 (corresponding to the ectodomain with the preceding hydrophobic leader sequence and an upstream insect Kozak sequence ( CCTATAAAT) plus a C-terminal Factor Xa site and a 6xHis tag) was subcloned into the BamHI site of the dicistronic baculovirus transfer vector pAcUW31 (BD Biosciences Clontech, Mountain View, California, United States). The ectodomain was truncated after residue Gly299 (Gly275 using numbering corresponding to mature class I MHC heavy chains). cDNA encoding human β2m plus its hydrophobic leader sequence was inserted into the BglII site of the transfer vector. Recombinant baculovirus was generated by co-transfection of the transfer vector with linearized viral DNA (Baculogold; BD Biosciences Clontech) and supernatants were harvested from baculovirus-infected Tn5 (High Five) insect cells. Cell supernatants were buffer exchanged into 20 mM Tris (pH 7.4)/150 mM NaCl, and concentrated to 1 liter using an Amicon RA2000 tangential-flow concentrator (Amicon, Beverly, Massachusetts, United States) with a 10 kDa cutoff membrane (Pall Corporation, East Hills, New York, United States). The resulting solution was adjusted to 50 mM Tris (pH 7.4)/300 mM NaCl/10 mM imidazole/10% glycerol, and loaded onto an 8-ml Ni-NTA column (Qiagen, Valencia, California, United States) equilibrated in 50 mM Tris (pH 7.4)/300 mM NaCl/10% glycerol. The column was washed with a similar buffer containing 40 mM imidazole and the bound M10.5 eluted in 250 mM imidazole. M10.5-containing fractions were pooled, concentrated to 2 ml, and loaded onto a 16/60 Superdex 75 column (Amersham Biosciences Corp., Piscataway, New Jersey, United States) equilibrated in 20 mM Tris (pH 7.4)/150 mM NaCl/1 mM EDTA/1 mM β-mercaptoethanol. SDS-PAGE analysis of the major peak on the gel filtration column revealed two bands migrating with apparent molecular masses of 37 kDa (expected mass 32.6 kDa + carbohydrate), corresponding to the M10.5 ectodomain, and 12 kDa (expected mass 11.7 kDa), corresponding to β2m. The M10.5-β2m peak eluted at a position corresponding to a protein of approximately 47 kDa, suggesting that the protein is monomeric (i.e., a single heterodimer). Fractions containing M10.5 were pooled resulting in a yield of approximately 1.5 mg per liter of insect cell supernatant.

N-terminal sequencing of the M10.5 heavy chain was accomplished by first blotting gel-run protein to a PVDF membrane (polyvinylidene fluoride, Millipore, Billerica, Massachusetts, United States), followed by sequencing using a 492 cLC Procise protein micro-sequencer (Applied Biosystems, Foster City, California, United States). The sequence obtained, SHWLKT, corresponds to the processed mature form of M10.5. In our numbering system, the first amino acid of the processed M10.5 heavy chain is denoted as residue 2 to correspond with the numbering of the processed forms of classical class I MHC molecules.

A vector for expression of M10.5 in CHO cells was constructed by subcloning the analogous region of M10.5 into a derivative of pBJ5-GS, which carries the glutamine synthetase gene as a selectable marker and means of gene amplification in the presence of methionine sulfoximine [62]. The M10.5 expression vector was transfected into CHO cells together with a human β2m expression vector as described [33]. Selection, amplification, maintenance of methionine sulfoximine-resistant cells, and identification of M10.5-expressing cells were done as described [46]. M10.5-β2m heterodimers were purified from CHO cell supernatants as described for the insect cell-derived protein.

### Crystallization and data collection

Crystals (space group P2_1_2_1_2_1_; *a* = 124.11 Å, *b* = 124.71 Å, *c* = 149.37 Å; five molecules per asymmetric unit) were grown at room temperature utilizing the hanging drop method by combining 1 μl of protein solution (approximately 8 mg/ml of insect cell-derived M10.5) with 1 μl of precipitant containing 0.1 M imidazole (pH 8.0)/20% PEG 1000/0.2 M calcium acetate. Crystals were cryopreserved in liquid nitrogen after soaking in mother liquor containing 10% glycerol as a cryoprotectant. Native datasets were collected at 100 K at beamline 12.3.1 at the Advanced Light Source (Berkeley, California, United States) and beamline 9–2 at the Stanford Synchrotron Radiation Laboratory (Stanford, California, United States). The data were indexed and scaled using the HKL suite of programs (HKL Research, Charlottesville, Virginia, United States) (see Table 1). The statistics reported in Table 1 refer to a single merged native dataset obtained by including frames from both native datasets in the scaling procedure.

### Structure solution and refinement

A protein–protein BLAST search indicated that Qa-2 and H-2D^d^ are the closest-related proteins in sequence to M10.5 for which crystal structures are available. Molecular replacement was carried out using the CCP4 program AMORE [63,64] with an all atom version of Qa-2 (not including the bound peptide) as a search model. Molecular replacement was not successful using each dataset individually, but a solution was obtained when the data were merged into a single native dataset (see Table 1). Five molecules were located in the asymmetric unit giving an *R*-factor of 47.9% after rigid-body refinement.

The model was refined using all reflections to 3.0 Å, but as the data are only approximately 60% complete between 3.1 Å and 3.0 Å, the effective resolution of the structure is approximately 3.2 Å. Test set reflections (5% of total) were picked using the thin shell method in DATAMAN [65] to reduce the influence of NCS correlations on *R-*factor calculations. To reduce the possibility of model bias, initial averaged maps were generated using the Qa-2 structure (open groove) or the FcRn structure (closed groove) to generate model structure factors. Maps produced by both methods indicated an open M10.5 groove. The M10.5 model was built using the program O [66] into real space–averaged, annealed composite omit electron-density maps in which 5% of the molecule was omitted at a time. Simulated annealing and grouped B-factor refinement with 5-fold NCS constraints was carried out using CNS [67]. Once the *R*_free_ value stopped improving with successive cycles of refinement, the NCS constraints were relaxed to NCS restraints using a weight of 300 kcal/mol·Å^2^. Parts of the model that significantly deviated between NCS-related molecules or that formed packing contacts were removed from the NCS restraints. The model stereochemistry was checked after each round of refinement using PROCHECK [68] and WHAT_CHECK [69]. The model (*R*_cryst_ = 26.6%; *R*_free_ = 30.3%) includes 264 out of 274 residues in the M10.5 heavy chain and all 99 residues of β2m (Table 1). Residues 145–150 and 195–198 are not seen in the electron-density map, and 36 sidechains in the molecule 1 M10.5 heavy chain and 13 sidechains in β2m are disordered and were modeled as alanines. The average main chain temperature factors per domain for molecule 1 are 48.3 Å^2^ (α1–α2), 68.2 Å^2^ (α3), and 44.4 Å^2^ (β2m). The temperature factors are the highest in the distal half of the α3 domain, correlating with this region having less continuous electron density than other regions of the maps.

Groove surface area calculations were performed as previously described [30,33] using the GRASP program [42]. Alignments of the M10.5 α1- α2 domains with other α1–α2 platforms were carried out with the CCP4 program Lsqkab [63] using platform β-sheet residues 3–13, 21–28, 34–37, 46–47, 93–103, 111–118, 122–126, and 133–135. Figures were produced using Molscript [70], Raster3D [71], and PyMOL [72].

### Thermal stability analyses

An AVIV 62A DS CD spectrometer with a thermoelectric cell holder and a cuvette with a 1-mm path length was used to monitor heat-induced unfolding of insect cell-derived M10.5 using samples containing 15 μM protein in 50 mM phosphate buffer. In one experiment, the protein solution also contained 20% polyethylene glycol 1000. The CD signal was monitored at 223 nm while the temperature was increased from 1 to 100°C in 1 degree increments with an equilibration time of 2 min and an averaging time of 30 s. *T*_m_s were determined by estimating the half-point of the ellipticity change between the pure native and pure denatured states.

### Acid elutions and peptide sequencing

Samples of purified M10.5 expressed in CHO and insect cells were analyzed for the presence of bound peptides as previously described [46]. In these experiments, FcRn (or HFE in an independent experiment; data not shown) served as a negative control since it had been previously established by biochemical and crystallographic methods that FcRn and HFE do not associate with endogenous peptides [32,33,47]. UL18 served as the positive control, because it associates with endogenous peptides when expressed in CHO cells [46,52]. A 20-fold molar excess of the H-2K^d^–binding peptide SYIPSAEKI [73,74] was added to insect cell M10.5 followed by gel-filtration chromatography to remove unbound peptide. This was repeated twice using a mixture of peptides, each at a 10-fold molar excess compared to M10.5. One mixture contained 13 peptides, including the following peptides used in a study reporting peptide-induced activation of VNO neurons [19]: AAPDNRETF (binds to H-2D^b^), AAPDARETA (mutated form of first peptide), ASNENMETM (binds to H-2D^b^), FAPGNYPAL (binds to H-2D^b^), SYFPEITHI (binds to H-2K^d^), SAFPEITHA (mutated form of preceding peptide), SYIPSAEKI (binds to H-2K^d^), SFVDTRTLL (binds to H-2K^d^), plus five other peptides: ALPHAILRL (binds to UL18) [52], TYCRTRALV (modified from an H-2K^d^–binding peptide) [51,74], RGYLYQGL (binds to H-2K^b^) [75], FAPGVFPYM (binds to H-2D^b^) [76], and ovalbumin-derived SIINFEKL (binds to H-2K^b^) [77]. The other mixture contained six of the above peptides. Acid elutions and sequencing were performed by established methods [51,78,79]. Briefly, 250 μg of CHO-derived and insect cell-derived soluble M10.5 (peptide-treated and untreated), UL18, and FcRn were concentrated to 100 μl in a Centricon 3 kDa cutoff centrifugal concentrator (Amicon). After addition of 1 ml of 50 mM ammonium acetate (pH 7.5), the proteins were again concentrated to 100 μl. This washing step was repeated once, followed by the addition of 1 ml of 10% acetic acid. The samples were heated to 70 °C for 15 min, and the solutions concentrated to 100 μl. This elution step was repeated once and the resulting 2 ml of filtrate was concentrated by evaporation to 50 μl. Automated Edman degradation was performed on 10 μl using a 492 cLC Procise protein micro-sequencer (Applied Biosystems). Analysis of acid eluates was also performed using a PerSeptive Biosystems Voyager Elite MALDI-TOF mass spectrometer (PerSeptive Biosystems, Framingham, Massachusetts, United States) with delayed extraction and a high sensitivity linear detector.

### Surface plasmon resonance binding assay

A Biacore 2000 biosensor system (Pharmacia-LKB Biotechnology, Uppsala, Sweden) was used to monitor interactions between M10.5 and potential ligands in mouse urine. Purified insect cell-derived M10.5 was coupled to two flow cells of a CM5 biosensor chip (Pharmacia-LKB Biotechnology) to coupling densities of 1600 and 4900 resonance units using standard amine coupling chemistry. Purified FcRn was coupled to a third flow cell to 1600 resonance units as a negative control, and a fourth flow cell was mock coupled. Urine was freshly collected from adult male and female C57BL/6 mice, and samples were frozen immediately. Male mouse urine (300–500 μl) was desalted through a G25 protein desalting spin column (Pierce Biotechnology, Rockford, Illinois, United States) prior to loading directly onto a RESOURCE Q (1 ml) high-performance ion exchange column (Amersham Biosciences). The column was then washed in 5 ml of buffer A (10 mM phosphate buffer [pH 7.0]), followed by elution in 10 ml of a linear gradient of buffer A to buffer B (10 mM phosphate buffer [pH 7.0]/300 mM NaCl) using a Vision Workstation HPLC system (Applied Biosystems). The concentration of MUPs in the HPLC fractions were estimated to be approximately 0.5 mg/ml based on Coomassie-stained SDS-PAGE gels and absorbance at 280 nm. Unfractionated urine contained approximately 100-fold higher concentrations of MUPs based on the gel staining. Whole and fractionated urine samples were diluted 10-fold into a buffer containing 50 mM HEPES (pH 7.4)/150 mM NaCl and injected at room temperature over the M10.5-, FcRn-, and mock-coupled flow cells. None of the samples showed any measurable responses aside from nonspecific refractive index changes, and no significant differences were observed between M10.5- and FcRn-coupled cells (data not shown). Although these results rule out binding between M10.5 and protein components in urine (e.g., MUPs, which are approximately 19 kDa), detection of molecules smaller than 1 kDa using a Biacore 2000 is problematic due to noise and buffer effects. As a positive control for the integrity of the coupled M10.5 proteins, a rabbit polyclonal antiserum raised against insect cell-purified M10.5 was injected over all four flow cells giving a high-affinity response only on the M10.5-coupled cells (data not shown).

## Supporting Information

Figure S1 Groove F_o_-F_c_ Electron-Density Maps for NCS-Related Molecules (A) Sigma-A–weighted F_o_-F_c_ maps contoured at 3.0 σ showing electron density within the α1–α2 domain. Electron density is shown for each of the five molecules in the asymmetric unit. There is no density indicative of bound peptide(s) or another type of groove occupant, except for a small peak in 5-fold averaged maps, which may result from approximately six carbons of a weakly interacting polyethylene glycol molecule in a location similar to that seen for a hexaethylene glycol molecule in the groove of Zn-α_2_-glycoprotein [27], which, like M10.5, was crystallized in the presence of polyethylene glycol. The peak could account for about half of a hexaethylene glycol molecule, but by contrast to the more ordered and stronger polyethylene glycol density in the Zn-α_2_-glycoprotein groove, the extra electron density within the M10.5 groove is weak and discontinuous. Thus if M10.5 does bind polyethylene glycol, it does not bind it in a single, ordered conformation, as observed in the structure of Zn-α_2_-glycoprotein [27], and probably does so to approximate a higher-affinity natural ligand.
(B) Real space–averaged F_o_-F_c_ map using the M10.5 model refined with 5-fold NCS constraints.
(2.0 MB JPG).

### Accession Numbers

Uniprot accession numbers (http://www.pir.uniprot.org) for proteins discussed in this paper are as follows: β2m, human (P61769), β2m, mouse (Q91XJ8), C receptor (Q9UNN8), CD1 (P11609), CD8 (P01731), FcRn (P13599), GABA_B_ (Q9WV18), Gα_i2_ (P08752), G_αo_ (P18872), H-2D^d^ (P01900), H-2K^b^ (P01901), H-2K^d^ (P01902), H2-M3 (Q31093), HFE (Q6B0J5), HLA-E (P13747), M10.5 (Q85ZW7), MIC-A (Q5C9P8), NKG2D receptor (P26718), Qa-2 (P79567), Rae-1β (O08603), T22 (Q9BCZ1), TAP1 (Q62427), TAP2 (P36371), UL18 (P08560), and Zn-α_2_-glycoprotein (P25311).

The Research Collaboratory for Structural Bioinformatics (RCSB) Protein Data Bank accession number (http://www.rcsb.org/pdb/) for the M10.5 structure is 1ZS8. The accession numbers for the other proteins discussed in this paper are as follows: FcRn (3FRU), H-2D^d^ (1BII), and Qa-2 (1K8D).

## Abbreviations

β2m: β2-microglobulin
CD: circular dichroism
CHO: Chinese hamster ovary
ER: endoplasmic reticulum
MALDI-TOF: matrix-assisted laser desorption ionization time-of-flight
MHC: major histocompatibility complex
MUPs: major urinary proteins
NCS: non-crystallographic symmetry
VNO: vomeronasal organ
